# Expression of Tight Junction Protein Claudin-1 in Human Crescentic Glomerulonephritis

**DOI:** 10.1155/2014/598670

**Published:** 2014-04-27

**Authors:** Ryo Koda, Atsunori Yoshino, Yuji Imanishi, Shinya Kawamoto, Yoshihiko Ueda, Eishin Yaoita, Junichiro James Kazama, Ichiei Narita, Tetsuro Takeda

**Affiliations:** ^1^Department of Nephrology, Dokkyo Medical University Koshigaya Hospital, No. 1-50, 2-Chome, Minami-Koshigaya, Koshigaya-shi, Saitama 343-8555, Japan; ^2^Department of Pathology, Dokkyo Medical University Koshigaya Hospital, No. 1-50, 2-Chome, Minami-Koshigaya, Koshigaya-shi, Saitama 343-8555, Japan; ^3^Department of Structural Pathology, Institute of Nephrology, Niigata University Graduate School of Medical and Dental Sciences, 1-757 Asahimachi-dori, Niigata 951-8510, Japan; ^4^Division of Clinical Nephrology and Rheumatology, Niigata University Graduate School of Medical and Dental Sciences, 1-757 Asahimachi-dori, Niigata 951-8510, Japan

## Abstract

The origin of crescent forming cells in human glomerulonephritis (GN) remains unknown. Some animal studies demonstrated that parietal epithelial cells of Bowman's capsule (PECs) were the main component of proliferating cells and PEC-specific tight junction protein claudin-1 was expressed in crescentic lesions. We investigated the expression of claudin-1 in human GN. Immunohistochemistry for claudin-1 was performed on 17 kidney biopsy samples with crescent formation. Colocalization of claudin-1 with intracellular tight junction protein ZO-1 was also evaluated by immunofluorescence double staining. Claudin-1 is expressed mainly at the cell to cell contact site of proliferating cells in cellular crescentic lesions in patients with these forms of human GN. Small numbers of crescent forming cells showed extrajunctional localization of claudin-1. Colocalization of claudin-1 with ZO-1 was found at cell to cell contact sites of adjacent proliferating cells. In control samples, staining of claudin-1 was positive in PECs, but not in podocytes. Our findings suggest that claudin-1 contributes to crescent formation as a component of the tight junction protein complex that includes ZO-1. Co-localization of claudin-1 with ZO-1 implies the formation of functional tight junction complexes in crescentic lesions to prevent the interstitial damage caused by penetration of filtered molecules from Bowman's space.

## 1. Introduction

Extracapillary proliferative cellular lesions or cellular crescents are a hallmark of severe inflammatory reactions in glomeruli and have been documented in various forms of glomerulonephritis (GN) [1]. Crescents are observed in various forms of GN including rapidly progressive GN, postinfectious GN, IgA nephropathy, purpura nephritis, and systemic lupus erythematosus. Though the origin of these crescentic lesions remains controversial, recent studies have clarified that not only inflammatory cells, but also intrinsic glomerular epithelial cells (i.e., parietal epithelial cells of Bowman's capsule (PECs) and glomerular epithelial cells or podocytes) contribute to the development of these crescents [2]. Smeets et al. demonstrated, using the genetic cell lineage tracing method, that PECs constitute the principal component of cellular crescents, especially at the early stage, in animal models of crescentic GN [3].

Claudin is a tetraspan-transmembrane protein and is a crucial component of tight junction complexes in epithelial and endothelial cells [4, 5]. Among the claudin subtypes, claudin-1 is expressed in the tight junctions of PECs in both murine models and human patients with GN and is therefore regarded as a marker of PECs [3, 6, 7].

Considering these observations, it is probable that PECs are the principal component of cellular crescents in various forms of human GN and that claudin-1 participates in the formation of cellular crescents by forming tight junctions among proliferating cells. We aimed to investigate the expression and localization of tight-junction protein claudin-1 in crescentic lesions of human GN.

## 2. Material and Methods

### 2.1. Subjects

Kidney biopsy samples from 17 patients with crescent formation were studied. Four kidney biopsy samples without crescent formation (minor glomerular abnormalities, minimal change nephritic syndrome, IgA nephropathy, and membranous nephropathy) and morphologically normal portions of kidneys from 5 patients with noninvasive renal tumors, who underwent nephrectomy, were used as control samples. Among those providing biopsy samples with crescent formation, five patients had IgA nephropathy, two had purpura nephritis, seven had myeloperoxidase (MPO) anti-neutrophil cytoplasmic antibody (ANCA) associated vasculitis, one had proteinase 3 (PR3-) ANCA associated vasculitis, one had anti-glomerular basement membrane (GBM) antibody associated rapidly progressive GN, and one had lupus nephritis. Kidney biopsy or nephrectomy was performed according to the routine diagnostic and therapeutic indication protocol used by Dokkyo Medical University Koshigaya Hospital. Acquired samples were either immediately fixed in formalin followed by embedding in paraffin, or snap- frozen at −80°C. Cellular crescents are defined as the presence of at least two cell layers in Bowman's space. The presence of crescents was identified by two nephrologists. Clinical features of patients included in this study are summarized in Table 1. All procedures in this study were conducted in accordance with the Declaration of Helsinki guidelines. The Institutional Review Board (IRB) of the Dokkyo Medical University Koshigaya Hospital approved this study (IRB approval number: 1206) and written informed consent was obtained from all participating patients.

### 2.2. Immunohistochemistry

Kidney biopsy specimens or tissue blocks from morphologically normal portions of surgically removed kidneys were immediately fixed with formalin and embedded in paraffin. Three *μ*m tissue sections were deparaffinized and rehydrated in a graded ethanol series. Antigen retrieval was performed by autoclaving for 10 min. Endogenous peroxidase activity was blocked with 3% hydrogen peroxidase and Protein Block Serum-Free (Dako, Japan) was applied to prevent nonspecific protein binding. Rabbit polyclonal antibody for claudin-1 (ab15098, Abcam, Cambridge, UK) or rabbit polyclonal anti-ZO-1 (Invitrogen) was diluted in Dako REAL Antibody Diluent (Dako) and used as a primary antibody (dilution of 1 : 200). Sections were incubated with primary antibody for 1 h at room temperature. Then, the slides were washed with phosphate buffered saline (PBS) three times and the sections were incubated with Histofine simple stain MAX-PO (MULTI) secondary antibody (Nichirei Bioscience, Japan) according to manufacturer's instructions. Counterstaining with hematoxylin was performed for nuclear staining.

### 2.3. Immunofluorescence

Three *μ*m cryosections were fixed in −20°C methanol for 10 min, washed with 7.4% PBS 3 times, treated with Protein Block Serum-Free for 30 min, and washed with PBS 3 times. Then, the samples were incubated with mouse monoclonal anti-claudin 1 antibody (1 : 100) (ab56417, Abcam) for 2 h at room temperature, washed with PBS 3 times, and incubated with Alexa Fluor 488 conjugated anti-mouse IgG (1 : 1000) (Cell Signaling Technology, Inc., Danvers, MA, USA) for 30 min. Again, the samples were washed with PBS 3 times, treated with Protein Block Serum-Free for 30 min, washed with PBS 3 times, and incubated with rabbit polyclonal anti-ZO-1 (1 : 400) (Invitrogen) at 4°C overnight. Samples were washed with PBS 3 times, and incubated with Alexa Fluor 555 conjugated anti-rabbit IgG (1 : 1000) (Cell Signaling Technology) for 30 min. Nuclear staining was performed using DAPI. Immunostaining for occludin was also performed using rabbit polyclonal anti-occludin antibody (Cat. 71-1500, Invitrogen, US) (1 : 200) and Alexa Fluor 555 conjugated anti-rabbit IgG. As a negative control, PBS was used instead of the primary antibodies. Immunofluorescence was examined with a laser scanning confocal microscope (OLYMPUS BX-51, Olympus, Tokyo, Japan).

### 2.4. Transmission Electron Microscopy

Small pieces of kidney biopsy specimens (approximately 0.5 to 1 mm^3^) were fixed in 2.5% glutaraldehyde in 0.05 M phosphate buffer (pH 7.4) overnight. The glutaraldehyde-fixed biopsy specimens were postfixed in 1% OsO_4_ in 0.05 M phosphate buffer for 30 min and embedded in epoxy resin. Ultrathin sections (approximately 100 nm) were prepared by cupper grids, then stained with uranyl acetate and lead citrate, and observed under a transmission electron microscope (JEM-1010, Nihon Densi, Tokyo, Japan).

### 2.5. Morphometric Analysis

Whole glomeruli were photographed in each biopsied sample with crescentic GN (*N* = 17). The border of the glomerulus and claudin-1 positive area was traced and the percentage of total claudin-1 positive area in the glomerulus was measured using ImageJ v. 1.47 software (http://rsb.info.nih.gov/ij). 2 × 5 mm size of tissue blocks from nephrectomized kidney were used as a control (*N* = 5). The difference between control sample and crescentic GN was analyzed by wilcoxon rank-sum test using Stata version 13 (Stata Corporation, College Station, TX). The level of statistical significance was set at *P* < 0.05.

## 3. Results

### 3.1. Immunohistochemistry

**Figure 1:**
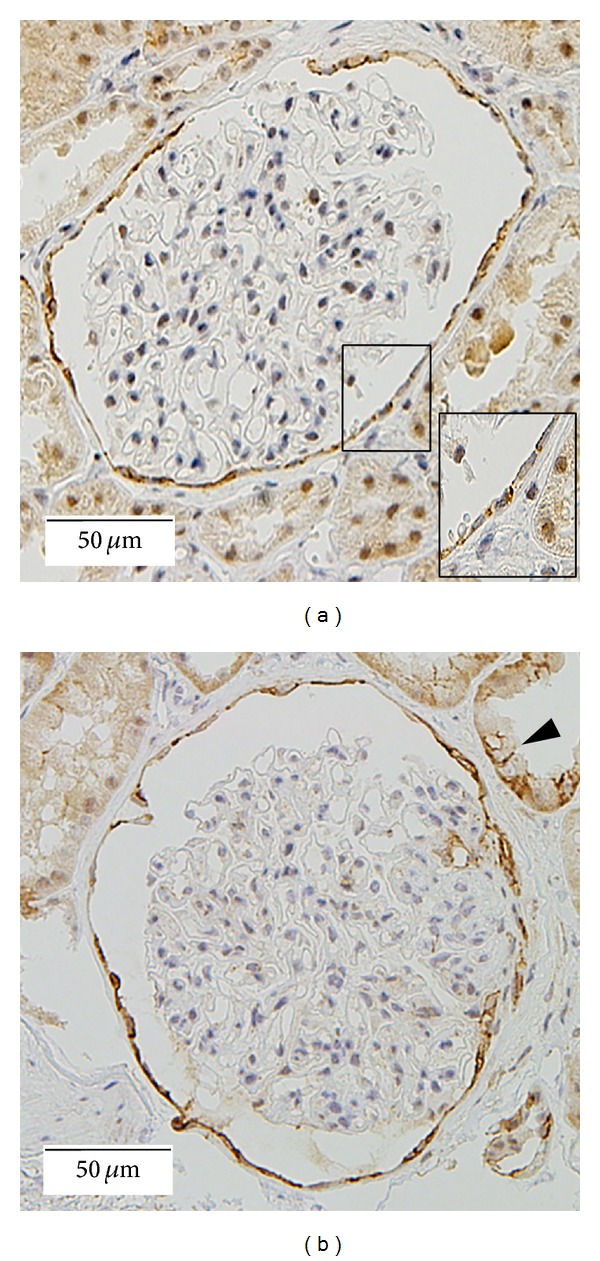
Claudin-1 stainings for nephrectomy ((a) ×200) and minimal change nephrotic syndrome ((b) ×200) samples are shown. In glomeruli, claudin-1 shows a strong, sharp staining pattern along cell membranes of adjacent PECs (magnified view in (a)). Staining signals are mainly detected at cell to cell contact sites. Distal tubules also express claudin-1 ((b) arrow head).

**Figure 2:**
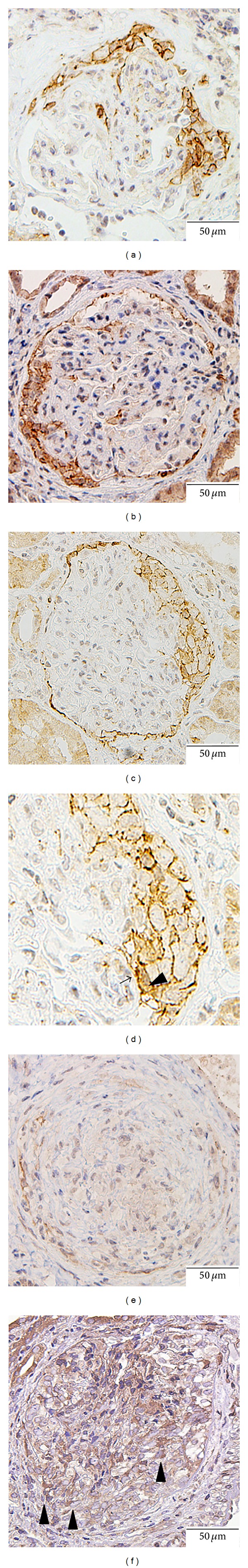
In cellular crescents, claudin-1 signals were mainly detected in cell membranes of adjacent proliferating cells (MPO-AAV ((a) ×200), IgA nephropathy ((b) ×200), lupus nephritis ((c) ×200 and (d) ×400). Extrajunctional cell membrane (arrow) and cytoplasmic (arrowhead) staining of claudin-1 are detected in some crescent forming cells ((d) arrowhead). Few claudin-1 positive cells were detected in necrotizing glomeruli ((e) ×200). ZO-1 positive cells were also detected in cellular crescent (arrow) ((f) ×200, sample from patient with MPO-AAV).

Claudin-1 showed a strong, sharp staining pattern along the cell membranes of adjacent PECs. In control samples, staining signals were mainly detected at cell to cell contact sites (Figures 1(a), and 1(b)). In cellular crescents, claudin-1 signals were mainly detected in the cell membranes of adjacent proliferating cells in all samples examined (Figures 2(a), 2(b), 2(c), and 2(d)). Occasionally, we observed cells to be positive for claidin-1 in the extrajunctional cell membrane and cytoplasm (Figure 2(d), arrow and arrowhead). No claudin-1 positive cells were detected in fibrous crescents or necrotizing glomeruli (Figure 2(e)). ZO-1 positive cells were also detected in cellular crescents (Figure 2(f), arrowheads).

### 3.2. Immunofluorescence

**Figure 3:**
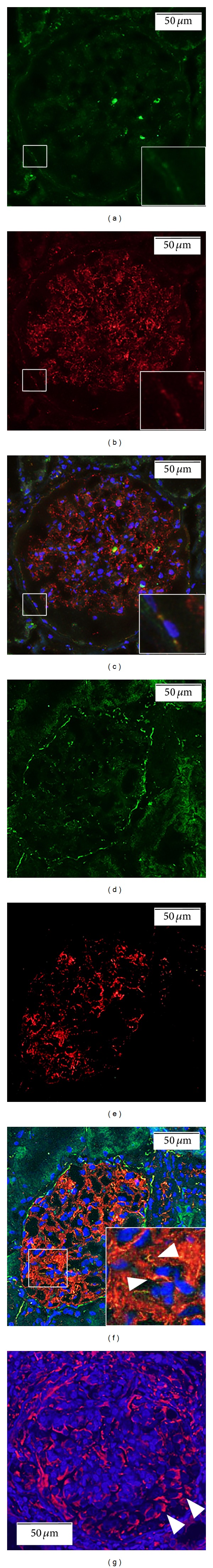
Immunofluorescence for claudin-1 (green) ((a), control and 3e, MPO-AAV) and ZO-1 (red) ((b) control and (e) MPO-AAV). Merged image ((c) control and (f) MPO-AAV) demonstrates that claudin-1 is expressed predominantly at cell to cell contact sites of adjacent PECs. Signals are distinctively concentrated in a dotted pattern in control sample (c). ZO-1, which is the major component of intracellular tight junction complexes, is visualized employing anti-ZO-1 antibody. Glomerular tufts also show positivity in ZO-1 staining. Colocalization of claudin-1 and ZO-1 is detected by confocal laser scanning microscopy (magnified view in (c) and (f)). In cellular crescents (f), lines that are double positive for anti-claudin-1 and anti-ZO-1 are observed (arrowhead); however, positivity of claudin-1 is less intense compared to ZO-1. Cell nuclei were stained with DAPI. (g) Occludin (red) is also expressed in crescentic lesion (arrowhead).

Claudin-1 is expressed exclusively at cell to cell contact sites of adjacent PECs in glomeruli without crescent formation (Figure 3(a)). Signals are distinctively concentrated in a dotted pattern. ZO-1 staining was observed in the tight junction complexes of adjacent PECs and foot processes of podocytes (Figure 3(b)). Claudin-1 and ZO-1 were colocalized (Figure 3(c)). In cellular crescents, claudin-1 expressing cells were minority (Figure 3(d)) compared to ZO-1 expressing cells (Figure 3(e)), but lines that were double positive for both anti-claudin-1 and anti-ZO-1 were observed (Figure 3(f)). Occludin, another fundamental intercellular component of tight junction complex, was also expressed in crescentic lesion (Figure 3(g)).

### 3.3. Transmission Electron Microscopy

**Figure 4:**
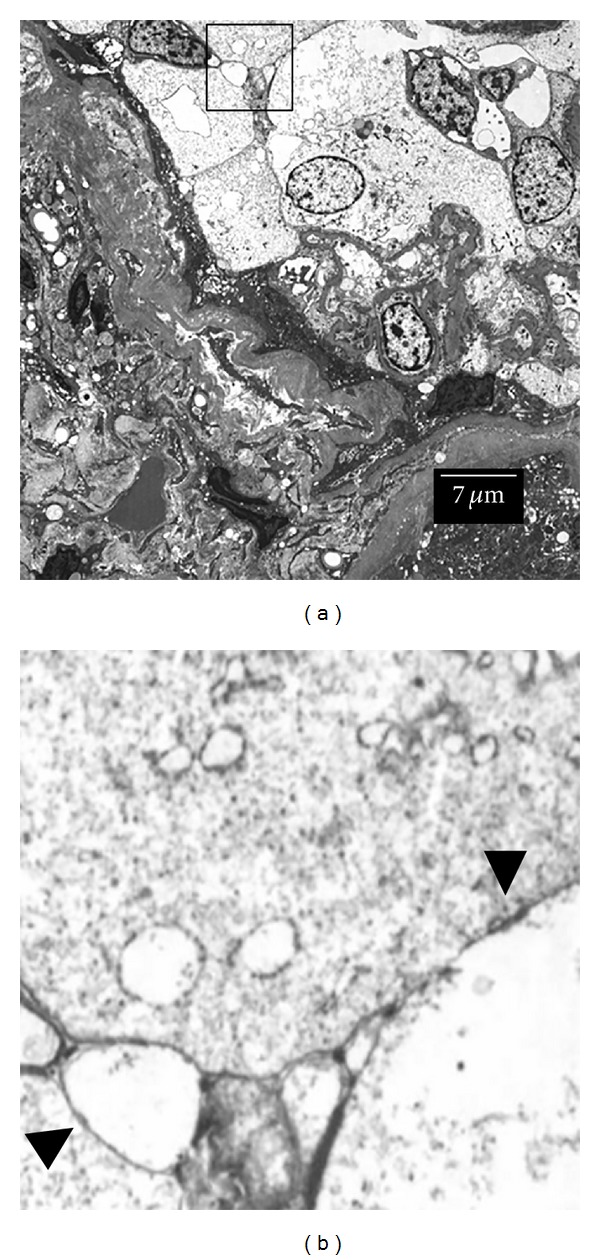
Transmission electron microscopy. Close contact between cell membranes of adjacent cells was observed, confirming the formation of tight junction in crescentic lesion in human glomerulonephritis ((b) arrowhead, sample from patient with IgA nephropathy). Figure (b) is magnified view of the framed area in (a).

Under electron microscopy, tight junction structure (close contacts between cell membranes of adjacent cells) was confirmed in crescent forming cells (Figure 4, biopsy sample from patient with IgA nephropathy).

### 3.4. Morphometric Analysis

**Figure 5:**
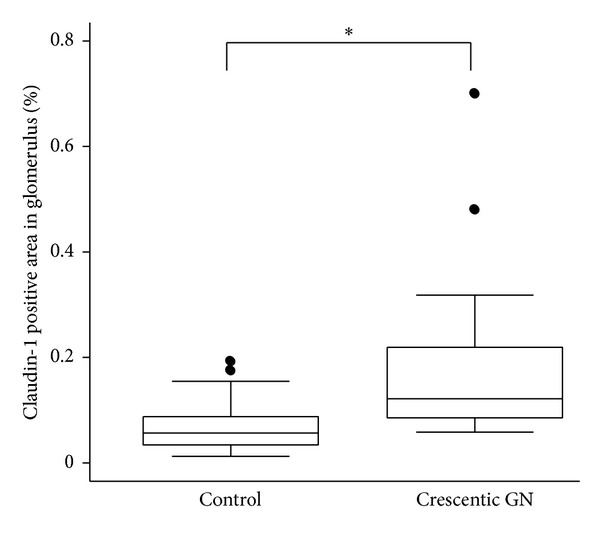
Percentage of claudin-1 positive area in glomerulus. Percentage of claudin-1 positive area in the glomerulus was measured using ImageJ software. The median percentages (interquartile range) of claudin-1 positive area in glomerulus of crescentic GN and control were 0.12 (0.85–0.22)% and 0.056 (0.34–0.88)%, respectively. *P < 0.01.

A total of 59 glomeruli in control samples (*N* = 5, nephrectomized sample) and 43 glomeruli in crescentic glomerulonephritis (*N* = 17) were evaluated. The median percentages (interquartile range) of claudin-1 positive area in glomerulus of crescentic GN and control were 0.12 (0.85–0.22) % and 0.056 (0.34–0.88) %, respectively (Figure 5). The difference between these two groups was statistically significant (*P* < 0.01).

## 4. Discussion

In the present study, we investigated the expression of claudin-1 and its colocalization with ZO-1 in various human glomerular diseases including IgA nephropathy, purpura nephritis, ANCA associated vasculitis (MPO-ANCA and PR-3 ANCA), anti-GBM-RPGN, and lupus nephritis. We obtained 3 important findings. First, claudin-1 was expressed mainly at cell to cell contact sites in proliferating cells in crescentic lesions. Second, nonjunctional mislocalization of claudin-1 was occasionally recognized in crescent forming cells, but not in PECs of control renal samples. Third, claudin-1 was colocalized with ZO-1, which is the fundamental intracellular component of tight junction complexes in epithelia [8], at the cell to cell contact sites of adjacent crescent forming cells.

Claudin-1 was initially discovered in 1998 by Furuse et al. as a 22 kD protein with four putative transmembrane domains [9]. Introduction of claudin-1 into fibroblasts leads to formation of tight junction strands in these intrinsically tight junction lacking cells [10]. Overexpression of claudin-1 in Madin-Darby canine kidney (MDCK) cells increases transepithelial electrical resistance and reduces paracellular flux, and claudin-1 is thus considered to contribute barrier function in epithelial cells [11]. Claudin-1 is crucial not only for tight junction formation, but also for intracellular entry of the hepatitis C virus [12] as a coreceptor of tetraspanin CD81 [13]. To date, at least 24 members of the claudin family, with tissue -specific distributions, have been identified in mice and humans. Expression patterns of claudin subtypes in nephron segments were precisely investigated in mice [14] and rabbits [15]. In glomeruli, claudin-1 [14, 15] and claudin-5 [16] were identified as tight junction proteins in PECs and podocytes, respectively. Kirk et al. performed an immunohistochemical analysis of claudin expression in human renal cortex and found that claudin-1 was expressed in the parietal epithelium of Bowman's capsule, distal convoluted tubule, and collecting duct [7].Smeets et al. demonstrated, using the genetic cell lineage tracing method, that, in a murine model of crescentic glomerulonephritis (induced by injection of anti-mouse nephrotoxic serum), the majority of the cell population in crescentic lesions is derived from PECS and that these lesions are positive for claudin-1 [3]. Their findings, taken together with our results, suggest that claudin-1 contributes to tight junction formation among adjacent proliferating cells in crescentic lesions in human glomerulonephritis.

We also found that expression of claudin-1 is not restricted to the cell to cell contact sites of adjacent cells. We detected claudin-1 staining in the nonjunctional cell membrane and cytoplasm of the cells composing the cellular crescents in crescentic glomerulonephritis. In immunofluorescence study, not all claudin-1 colocalized with ZO-1. These cells expressing nonjunctional claudin-1 might be the infiltrating immune cells, since the expression of claudin and other tight junction proteins in immune cells has been reported [17]. On the other hand, it is well known that expression of claudin is not restricted to tight junction strands in epithelial cells. Claudin-5 can be found at the apical membrane of the rat podocytes of puromycin aminonucleoside (PAN) induced nephrosis [16], and claudin-1 can be found in basolateral membrane of rat epididymis [18]. Various factors, such as interleukin-1*β* [19], helicobacter pylori [20], and dexamethasone [21], are known to affect the expression and localization of claudin. Moreover, Dhawan et al. demonstrated high expression of claudin-1 in human colorectal cancer tissues and that nuclear and cytoplasmic mislocalization of claudin-1 was frequently seen as compared to the normal mucosa [22]. They confirmed, by immunoblotting, expression of claudin-1 to predominate in the nuclear and cytoplasmic fractions of SW480 cells (derived from primary colorectal cancer) and SW620 cells (derived from a metastatic lesion) as compared to RIE cells (derived from noncancerous rat intestinal cells). They also demonstrated, using these two cell lines from human colorectal cancer, that modulation of claudin-1 expression resulted in the phenotypic change known as epithelial to mesenchymal transition (EMT) and suggested *β*-catenin signaling and E-cadherin expression to be possible mechanisms underlying these claudin-1 induced phenotypic changes. Mislocalization of junctional protein may participate in EMT, as Reichert et al. showed that in MDCK cells mutant ZO-1, which is composed of PDZ domains of ZO-1, localizes mainly in the cytoplasm, activates *β*-catenin signaling, and decreases epithelial marker expression [23]. Bariety et al. revealed that EMT occurs in the early phase in human pauci-immune crescentic glomerulonephritis [24]. Considering these observations, it is possible that nonjunctional claudin exists in human glomerular crescent and plays some novel functions. It is proposed that the nonjunctional cell membrane claudins might represent a reservoir for junctional claudins [25]. However, the role of claudins in the nonjunctional cell membrane and cytoplasm remains uncertain in this study. Recently, Ohse et al. established conditionally immortalized mouse glomerular parietal epithelial cells [26]. It might be useful to use a PEC-specific cell line to further clarify the roles of extrajunctional claudin-1 and related signaling pathways in crescent formation. This issue needs to be elucidated in future study.

ZO-1 is a 225 kD intracellular protein that is an indispensable component of tight junction complexes [8]. ZO-1 directly binds to the COOH-terminus of claudin-1 through its PDZ1 domain [27]. The PDZ1 domain of claudin-1 plays a pivotal role in the formation of normal tight junction complexes and in exerting selective molecular permeability, that is, barrier function [28]. In normal glomeruli, ZO-1 is expressed in the slit-diaphragms of podocytes [29]. In this study, we demonstrated colocalization of ZO-1 and claudin-1 in adjacent proliferating cells in human glomerular crescent. Actually, intensity of ZO-1 signal was stronger than claudin-1, and thus claudin-1 was partially colocalized with ZO-1 (Figure 3(f)). Wide variety of cells including immune cells and glomerular epithelial cells (i.e., PECs and podocytes) contribute to the formation of cellular crescent. Not only PECs but also podocytes and immune cells are known to express ZO-1 [17]. Though parietal epithelial cells that were positive in claudin-1 have been demonstrated to be the predominant population of the cellular crescent in the murine model of crescentic glomerulonephritis [3], it is unclear whether this is true in cellular crescent in human disease. ZO-1 positive and claudin-1 negative cells in human glomerular crescents could be the infiltrating immune cells or podocytes. The minority of claudin-1 positive cells in human glomerular crescent in our study might indicate that contribution of PECs to the formation of crescentic lesion is relatively small compared to murine models of crescentic GN. Ohse et al. proposed using low and high molecular weight tracers in experimental models of anti-GBM glomerulonephritis to show not only the basement membrane in Bowman's capsule, but also the tight junctions in PECs to possibly act as a second glomerular barrier preventing interstitial damage caused by penetration of filtered molecules from Bowman's space [30]. Thus, it is possible that formation of tight junction complexes with claudin-1 and ZO-1 in extracapillary proliferating lesions contributes to minimizing the solute-leakage induced interstitial damage by endowing crescents with a barrier property in human glomerulonephritis. Further experiments like permeability assay using PEC-specific cell line would contribute to verifying this hypothesis.

In conclusion, our results demonstrate that claudin-1 is expressed in cellular crescents in human glomerulonephritis. Claudin-1 is predominantly distributed at cell to cell contact sites of proliferating cells in crescentic lesions. Slight but significant distributions of this molecule in extrajunctional cell membrane and cytoplasm suggest a nonjunctional role of claudin-1. Colocalization of claudin-1 with ZO-1 raises the possibility of functional tight junction complex formation in crescentic lesions aimed at the prevention of the interstitial damage by penetration of filtered molecules from Bowman's space.

## Figures and Tables

**Table 1:** Clinical features of patients in this study.

No	Age (years)	Sex	Disease	Cr (mg/dL)	Hematuria (/*μ*L)	UP/UCr (g/gCre)	U-*β*2MG (*μ*g/L)	CRP (mg/dL)	Treatment
1	25	F	IgA nephropathy	10.4	381	3.8	31510	0.07	PSL, HD, LKT
2	57	M	IgA nephropathy	5.4	149	7.6	13185	0.35	mPSL, oral PSL
3	32	M	IgA nephropathy	0.9	140	2.5	95.6	0.11	mPSL, oral PSL
4	20	F	IgA nephropathy	1.0	60	8.2	48.6	0.06	mPSL, oral PSL
5	38	F	IgA nephropathy	1.7	1708	3.4	9378.5	5.09	mPSL, oral PSL
6	20	F	Purpura nephritis	0.7	162	1.1	182.1	0.06	None
7	39	F	Purpura nephritis	0.7	212	2.1	74.2	0.06	Oral PSL
8	63	M	MPO-AAV	9.0	140	0.5	22370	3.43	mPSL, oral PSL
9	39	M	MPO-AAV	13.1	208	3.8	226.8	13.37	mPSL, oral PSL
10	61	M	MPO-AAV	2.9	130	0.8	8471.9	2.82	mPSL, oral PSL
11	68	M	MPO-AAV	2.3	1030	3.8	3064.3	3.83	mPSL, oral PSL
12	77	M	MPO-AAV	3.1	243	3.3	90.6	5.73	mPSL, oral PSL
13	70	M	MPO-AAV	2.3	193	1.3	164.9	5.37	mPSL, oral PSL
14	71	M	MPO-AAV	9.1	457	0.4	47683	2.75	mPSL, oral PSL
15	64	M	PR3-AAV	2.8	285	1.7	523.3	23.55	mPSL, CY
16	64	M	Anti-GBM RPGN	15.4	420	2.4	70121	6.40	mPSL, PE, HD
17	63	F	Lupus nephritis (IV + V)	1.3	281	7.0	337.2	2.03	mPSL

Cr: creatinine; CY: cyclophosphamide; GBM: glomerular basement membrane; HD: hemodialysis; IgA: immunoglobulin A; LKT: living kidney transplantation; MPO-AAV: myeloperoxidase anti-neutrophil cytoplasmic antibody associated vasculitis; mPSL: methyl-prednisolone pulse therapy; PE: plasma exchange; PR3-AAV: proteinase 3 anti-neutrophil cytoplasmic antibody associated vasculitis; PSL: prednisolone; RPGN: rapidly progressive glomerulonephritis; UP/UCr: urine protein to creatinine ratio; U-*β*2MG: urinary beta2-microglobulin.
